# Improved Performance of Ionic Liquid Supercapacitors by using Tetracyanoborate Anions

**DOI:** 10.1002/celc.201701164

**Published:** 2018-01-04

**Authors:** Vitor L. Martins, Anthony J. R. Rennie, Nedher Sanchez‐Ramirez, Roberto M. Torresi, Peter J. Hall

**Affiliations:** ^1^ Chemical and Biological Engineering University of Sheffield, Sir Robert Hadfield Building Mappin Street Sheffield S1 3JD England, UK; ^2^ Depto. Química Fundamental, Instituto de Química Universidade de São Paulo Av. Prof. Lineu Prestes 748 05508-000 São Paulo, SP Brazil

**Keywords:** tetracyanoborate, ionic liquids, electrochemical double-layer capacitors, supercapacitors

## Abstract

Supercapacitors are energy storage devices designed to operate at higher power densities than conventional batteries, but their energy density is still too low for many applications. Efforts are made to design new electrolytes with wider electrochemical windows than aqueous or conventional organic electrolytes in order to increase energy density. Ionic liquids (ILs) with wide electrochemical stability windows are excellent candidates to be employed as supercapacitor electrolytes. ILs containing tetracyanoborate anions [B(CN)_4_] offer wider electrochemical stability than conventional electrolytes and maintain a high ionic conductivity (6.9 mS cm^−1^). Herein, we report the use of ILs containing the [B(CN)_4_] anion for such an application. They presented a high maximum operating voltage of 3.7 V, and two‐electrode devices demonstrate high specific capacitances even when operating at relatively high rates (ca. 20 F g^−1^ @ 15 A g^−1^). This supercapacitor stored more energy and operated at a higher power at all rates studied when compared with cells using a commonly studied ILs.

Supercapacitors, or electrochemical double‐layer capacitors (EDLCs), are energy storage devices that are designed to operate at higher power densities than conventional batteries. EDLCs rely mainly on the charge stored in the double‐layer formed at polarized electrode/electrolyte interfaces, so that the interactions of ions and electrode surface play a crucial role in the charge storage process. However, one consequence of this storage mechanism is that EDLCs have much lower energy densities than batteries, which limits their engineering applications.^1^, ^2^, ^3^, ^4^

The energy in EDLC is given by *E*=*C* ⋅ (*V*)^2^/2, *E* is the gravimetric or volumetric specific energy (J g^−1^ or J m^−3^) and *C* is the specific capacitance (F g^−1^ or F m^−3^). *V* (V) is the (operationally defined) maximum operating voltage, i. e. the maximum polarization voltage at which EDLCs can operate with tolerable loss of capacitance. Consequently, electrolytes that maximize the operating voltage are attractive because they maximize specific energy. In general, ionic liquids (ILs) operate at higher voltage than conventional aqueous or organic electrolytes and therefore fulfil this requirement. IL based EDLCs have not been developed commercially because they possess lower ionic conductivity values than conventional electrolytes, leading to higher internal resistance which results in reduced power density.^5^, ^6^, ^7^, ^8^, ^9^, ^10^

Over the past two decades, myriad ILs have been synthesized and characterized for use as EDLC electrolytes, reviewed by Salanne.^8^ The ideal IL will maximize electrochemical stability, thermal stability, ionic conductivity and possess a liquid range to satisfy the engineering application requirements. *N*‐butyl‐*N*‐methylpyrrolidinium bis(trifluoromethylsulfonyl)imide ([Pyr_1,4_][Tf_2_N]) is one of the most commonly researched ILs because it possesses a good balance between these desirable properties^11^, ^12^, ^13^, ^14^, ^15^, ^16^, ^17^, ^18^, ^19^, ^20^, ^21^, ^22^ and acts as an informal reference against which other ILs can be compared.

The relative advantages and disadvantages of using ILs compared to organic electrolytes can be seen in Table 1. For example, [Pyr_1,4_][Tf_2_N] possess a maximum operating voltage of 3.7 V compared to 3.0 V for PC based electrolytes. Different operating voltages for organic solvents based electrolytes can be found in literature, since it greatly depends on electrode materials employed. Considering a constant capacitance, the difference in voltage mentioned above would give an increase in specific energy density of over 50 %. In addition, ILs have higher thermal stability which is a great advantage. However, the downside of using [Pyr_1,4_][Tf_2_N] is that its ionic conductivity is 2.7 mS cm^−1^, much lower than 12.2 mS cm^−1^ for PC based electrolyte. This would result in a much lower power density.^2^

**Table 1:** Physicochemical properties of PC‐based electrolyte, [Pyr_1,4_][Tf_2_N] and ILs containing anions with cyano group at 25 °C and thermal decomposition.

	*T*_d_ [°C]	*ρ* [g cm^−3^]	*η* [mPa s]	*σ* [mS cm^−1^]	*V* [V]	Ref.
1 mol L^−1^ TEABF_4_/PC	–^[a]^	1.19	3.72	12.2	3.0	2,28,29
[Pyr_1,4_][Tf_2_N]	445	1.40	78.0	2.7	3.7	13,20
[Pyr_1,4_][N(CN)_2_]	283	1.01	30.8	12.0	2.5	19,30,31
[Pyr_1,4_][C(CN)_3_]	346	1.01	29.0	8.7	2.9	20
[Pip_1,4_][C(CN)_3_]	352	1.01	57.8	4.2	3.0	20
[Pyr_1,4_][B(CN)_4_]	403	0.97	47.9	6.9	3.7	27, this work
[Pip_1,4_][B(CN)_4_]	414	0.97	88.5	3.7	3.7	27,32, this work

[a] PC: flash point: 116 °C; boiling point: 240 °C.

Wolff *et al*.^19^ have shown that is possible to increase the power density by using ILs containing the dicyanamide anion ([N(CN)_2_]). Table 1 shows that the ionic conductivity of [Pyr_1,4_][C(CN)_2_] is 12 mS cm^−1^, which is much larger than the [Pyr_1,4_][Tf_2_N] analogue and comparable to PC based electrolyte. However, the determined operating voltage is only 2.5 V which is much lower than the 3.7 V used for [Pyr_1,4_][Tf_2_N].^19^ In an earlier work,^20^ we have studied the tricyanomethanide ([C(CN)_3_]) anion and found similar results, obtaining higher power but reduced energy densities in comparison with [Pyr_1,4_][Tf_2_N].^20^

The operating voltage usually depends on the cationic resistance to reduction and the anionic resistance to oxidization,^23^ nevertheless, it has been found that both cations and anions play important roles in the definition of positive and negative limits.^24^ Using molecular modelling, Dhungana et al.^25^ have shown that in the IL 1‐ethyl‐3‐methylimidazolium tetracyanoborate [Im_1,2_][B(CN)_4_], the cation is more likely to oxidize than the anion, resulting in an IL with greater electrochemical stability than other ILs with cyano based anions.^25^ Lust *et al*. investigated the use of this IL and show that the anion composition and its charge distribution have significant influence on resultant power densities.^18^ Moreover, [B(CN)_4_] anions may undergo polymerization at high overpotentials, thereby acting as a safety mechanism.^26^ ILs containing [B(CN)_4_] anions are known to possess a wide electrochemical stability window when an inert electrode material, (such as glassy carbon)^27^ is employed. However, it is important to determine an ILs stability when combined with the activated carbon material used in EDLC electrodes, which presents much higher surface area and can catalytically decrease the ILs electrochemical stability. In this work, we present the results of EDLCs containing two ILs based on tetracyanoborate anions: *N*‐butyl‐*N*‐methylpiperidinium ([Pip_1,4_][B(CN)_4_]) and [Pyr_1,4_][B(CN)_4_], shown in Figure 1a (the physicochemical characteristics of these ILs are described elsewhere^27^).

**Figure 1:**
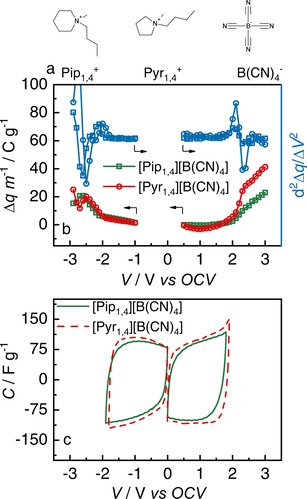
a) Chemical structure of the cations [Pip_1,4_] and [Pyr_1,4_], and the anion [B(CN)_4_]. b) Maximum operating voltage determination. The left axis shows evolution of *q*_charge_‐*q*_discharge_ for positive and negative scans in different windows when using the ILs [Pip_1,4_][B(CN)_4_] (green squares) and [Pyr_1,4_][B(CN)_4_] (red circles). The right axis shows the second derivative of difference of charge for [Pip_1,4_][B(CN)_4_] (blue squares) and [Pyr_1,4_][B(CN)_4_] (blue circles). c) Cyclic voltammograms at the determined limits for [Pip_1,4_][B(CN)_4_] (green full line) and [Pyr_1,4_][B(CN)_4_] (red dashed line).

The method used to determine maximum operating voltage is based on the method proposed by Weingarth *et al*.^33^ and is described in detail elsewhere.^13^,^20^ Basically, the difference in coulombic charge between the discharge and charge processes (Δ*q*=*q*_charge_−*q*_discharge_) of cyclic voltammograms (CVs) recorded at 5 mV s^−1^ over different voltage windows were determined. The highest stability was chosen as the voltage when the d^2^Δ*q*/d*V* ^2^ exhibits a sharp increase, indicating a lower charge‐discharge efficiency due to electrolyte decomposition.

Figure 1b shows the maximum operating voltage determination for both ILs, [Pip_1,4_][B(CN)_4_] and [Pyr_1,4_][B(CN)_4_], from 0.5 V up to 3.0 V for positive determinations, and from −1.0 V down to −3.0 V for the negative determinations. A maximum operating voltage of 3.7 V was determined for both ILs which is much higher than the values found for other ILs containing anions based on the cyano group, such as [N(CN)_2_] (2.5 V)^19^ and [C(CN)_3_] (3.0 V)^20^, and similar to that shown by the IL [Pyr_1,4_][Tf_2_N].^12^ It is worth noting that other approaches to enhance the maximum operating voltage of EDLCs were also described in literature. For example, Shen and Hu^34^ used carbon black in the positive electrode and activated carbon in the negative electrode and obtained a device operating at 3.0 V using conventional electrolyte (1 mol L^−1^ TEABF_4_ in PC), however, the lower surface area of carbon black compared to activated carbon decreases the overall device specific capacitance. Moreover, Balducci and collaborators^35^, ^36^, ^37^, ^38^ have employed different organic solvents or ILs as conductive salts in organic solvent to increase the electrolyte stability. A maximum operating voltage of 3.5 V was obtained when 1 mol L^−1^ of [Pyr_1,4_][Tf_2_N] in PC^35^ or TEABF_4_ in 3‐cyanopropionic acid methyl ester were used.^36^ The [B(CN)_4_] ILs still show greater maximum operating voltage than these alternatives.

The CVs at the determined limits can be seen in Figure 1c, they show the expected rectangular shape for capacitor behavior and no faradaic peaks are observed. [Pip_1,4_][B(CN)_4_] has 0.1 V higher stability on the negative side than the [Pyr_1,4_][B(CN)_4_], however it is also 0.1 V lower on the positive side; thus, overall, they have the same operating voltage. To ensure charge distribution is symmetrical for both electrodes in the EDLCs when operating at high rates (since 3.7 V at 5 mV s^−1^ would discharge in 740 s), the ratio of the charge quantities in the discharge steps of negative (*q*_‐_) and positive (*q*_+_) scans were analyzed at different scan rates at the determined window for each electrode (positive and negative).^39^ Figure S1 (in Supporting Information (SI)) shows the mass ratio of positive to negative electrode required to balance the charge (*m*_+_/*m*_−_=*q*_−_/*q*_+_). This ratio increases with scan rate, and in order to have an extended life the mass ratio at 100 mV s^−1^ was used for EDLC design which was determined to be 2.2 for EDLCs containing [Pip_1,4_][B(CN)_4_] and 1.8 for EDLCs containing [Pyr_1,4_][B(CN)_4_] or [Pyr_1,4_][Tf_2_N] (not shown, but it was considered negative and positive limits of −2.0 and 1.7 V, as a total of 3.7 V as the [B(CN)_4_] analogues).

Figure 2a shows CVs at 5 mV s^−1^ obtained using the EDLCs containing the ILs [Pip_1,4_][B(CN)_4_] and [Pyr_1,4_][B(CN)_4_] as electrolytes from 0 to 3.7 V. They have similar rectangular shapes, indicating that no faradaic reactions occur. Coulombic efficiencies higher than 98 % were observed in CVs, indicating the high degree of reversibility with respect to the amount of charge stored. As in the maximum operating voltage determination, it is observed that the EDLC containing [Pyr_1,4_][B(CN)_4_] has a higher capacitance than the [Pip_1,4_] counterpart. This is a likely consequence of its higher ionic conductivity (6.9 *vs* 3.7 mS cm^−1^, see Table 1) and smaller cation size; these features combined facilitate mass transport into the smaller pores, resulting in an increased specific capacitance. It is worth noting that this feature was also observed when the [C(CN)_3_] anion was used with the same cations.^20^ Figure 2b shows that higher capacitance is retained over a broad range of scan rates when the [Pyr_1,4_][B(CN)_4_] IL is used. In addition, as the scan rate increases the degree of retention is even greater, for instance, at 200 mV s^−1^, the [Pyr_1,4_][B(CN)_4_] EDLC retains 70 % of its initial capacitance while [Pip_1,4_][B(CN)_4_] EDLC retains only 53 %. Moreover, [Pyr_1,4_][B(CN)_4_] EDLC has specific capacitance of *ca*. 20 F g^−1^ at 200 mV s^−1^ which is similar to that displayed by [Pip_1,4_][B(CN)_4_] EDLC at 25 mV s^−1^.

**Figure 2:**
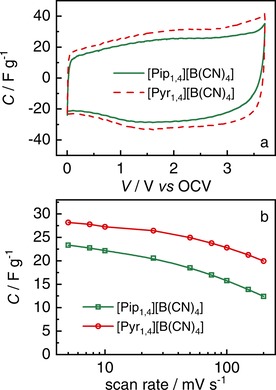
a) Cyclic voltammograms of EDLCs containing [Pip_1,4_][B(CN)_4_] (green full line) and [Pyr_1,4_][B(CN)_4_] (red dashed line) from 0 to 3.7 V at 5 mV s^−1^. b) Discharge specific capacitance of EDLCs containing [Pip_1,4_][B(CN)_4_] (green squares) and [Pyr_1,4_][B(CN)_4_] (red circles) determined at different scan rates.

Galvanostatic charge‐discharge (GCD) cycling was also performed. EDLCs were cycled 20 times at each current rate from 0.5 to 10 A g^−1^ (15 A g^−1^ for [Pyr_1,4_][B(CN)_4_]). Figure 3a–b show the 20^th^ cycle profile for each GCD rate; results using [Pyr_1,4_][Tf_2_N] are also illustrated for comparison. The three ILs present the linear profile expected for EDLCs and, as with CV experiments, [Pyr_1,4_][B(CN)_4_] outperforms [Pip_1,4_][B(CN)_4_] and also the [Tf_2_N] analogue operating at the same voltage of 3.7 V. Figure 3c shows the specific capacitance calculated from the 20^th^ GCD cycles. As anticipated by the GCD profiles, [Pyr_1,4_][B(CN)_4_] retains more capacitance at higher currents, showing a specific capacitance of *ca* 20 F g^−1^ at 15 A g^−1^, while [Pip_1,4_][B(CN)_4_] and [Pyr_1,4_][Tf_2_N] resulted in an excessive *iR* drop at such a high rate (i. e. the *iR* drop was greater than the operating voltage). The equivalent series resistance (ESR) presented in Figure 3d was calculated at each rate from the *iR* drop in the discharge process considering the following relationship: *ESR*=Δ*E*/(2 ⋅ *i*) where Δ*E* is the voltage drop after 40 ms from the change in current and *i* is the discharge current. At 10 A g^−1^, [Pip_1,4_][B(CN)_4_], [Pyr_1,4_][B(CN)_4_] and [Pyr_1,4_][Tf_2_N] have ESRs of 17.3, 10.5 and 16.7 Ω respectively. The lower viscosity and higher ionic conductivity presented by [Pyr_1,4_][B(CN)_4_] results in a device with lower ESR, while the other two liquids produce similar ESRs as they also have similar viscosity and ionic conductivity, as showed in Table 1. As mentioned above, only [Pyr_1,4_][B(CN)_4_] EDLC delivered any energy at 15 A g^−1^, and it is worth noting that the ESR increased to 15.2 Ω, which is still lower than the other EDLCs at lower rates. This sudden increase indicates that the energy storage process must be close to the mass transfer limit, raising the ESR. It is worth noting that EDLCs containing the same electrode composition and 1 mol L^−1^ TEABF_4_ in PC as electrolyte showed an ESR of 16.3 Ω, i. e. lower than [Pip_1,4_][B(CN)_4_] and [Pyr_1,4_][Tf_2_N] but higher than [Pyr_1,4_][B(CN)_4_], consolidating the superior performance of the latter. The cell design clearly needs optimization in order to decrease the high ESR found in these EDLCs compared to values found in literature, but the comparison among the devices showed in this study support the superior performance of EDLC containing [Pyr_1,4_][B(CN)_4_] as electrolyte.

**Figure 3:**
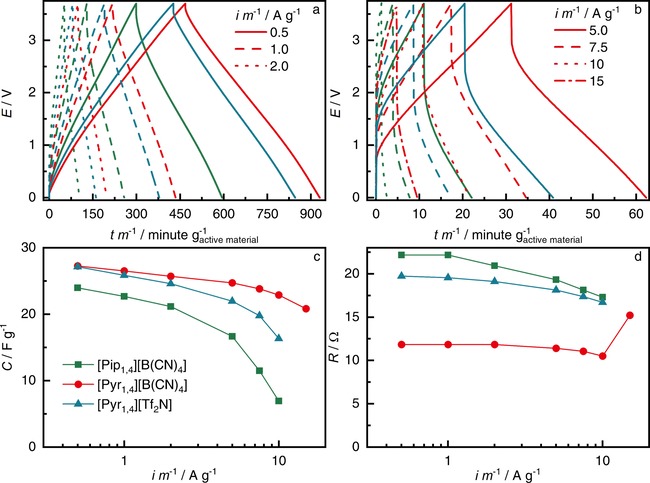
a,b) GCD at different rates for EDLCs containing [Pip_1,4_][B(CN)_4_] (green full line), [Pyr_1,4_][B(CN)_4_] (red dashed line) and [Pyr_1,4_][Tf_2_N] (blue dotted line). c) Discharge specific capacitance and d) ESRs calculated from the 20^th^ GCD for EDLCs containing [Pip_1,4_][B(CN)_4_] (green squares), [Pyr_1,4_][B(CN)_4_] (red circles) and [Pyr_1,4_][Tf_2_N] (blue triangles).

In order to better understand the behavior of ILs in EDLCs, electrochemical impedance spectroscopy (EIS) was performed on the devices at their rest voltage. Figure S2 (in SI) shows the Nyquist plot for cells using the two ILs containing [B(CN)_4_] and includes [Pyr_1,4_][Tf_2_N] for comparison. The three spectra show capacitive behavior at low frequencies with spectra being almost parallel to the axis. A semi‐circle is observed in the high frequency region for the three ILs, the electrolyte resistance (*R_s_*) can be observed where the semi‐circle crosses the real axis. The *R_s_* are in good agreement with the ionic conductivities of the ILs, since [Pyr_1,4_][B(CN)_4_] exhibits the highest ionic conductivity and the lowest *R_s_* (2.9 Ω), while [Pip_1,4_][B(CN)_4_] and [Pyr_1,4_][Tf_2_N] present very similar *R_s_* (5.6 and 5.7 Ω, respectively). The depressed semi‐circle is a consequence of the impedance distribution in the carbon pores with different microstructure^18^ and possibly due to heterogeneous adsorption of ions in the carbon surface being a determinant step at high frequencies.^40^ Other possibilities for the observed high frequency shape are the formation of a passivating film on the current collector (i. e. the stainless‐steel coin cell spacer) or an increased resistance between electrodes and electrolyte/separator.^41^ Nonetheless, the ionic resistance (*R*_i_, the semi‐circle diameter) observed for [Pip_1,4_][B(CN)_4_], [Pyr_1,4_][B(CN)_4_] and [Pyr_1,4_][Tf_2_N] are 5.7, 3.6 and 7.5 Ω, respectively. The resistances were found with the best fit of EIS data using the model R1‐(R2 CPE1)‐CPE2‐C1 (details are shown in Figure S2 and Table S1, in SI). The devices specific capacitances obtained from the following equation $C_{specific}=-1/2\pi f\cdot Z"\cdot m$ are 14.8 F g^−1^ for [Pip_1,4_][B(CN)_4_] and 18.1 F g^−1^ for both [Pyr_1,4_][B(CN)_4_] and [Pyr_1,4_][Tf_2_N], which is in agreement with the results from GCD since these two ILs also showed similar capacitance at low rate.

An alternative analysis of EIS data is the calculation of complex capacitance, so the energy stored can be evaluated by the real contribution ($C^{{}^{'}}=-Z"\left(\omega \right)/\left(\omega {\left|Z\left(\omega \right)\right|}^{2}\right)$ and the energy losses can be evaluated by considering the imaginary contribution ($C"=Z{}^{'}\left(\omega \right)/\left(\omega {\left|Z\left(\omega \right)\right|}^{2}\right)$ .^42^,^43^ Figure 4a–b show the real capacitance (*C′*) and the imaginary capacitance (*C′′*) as a function of frequency. The EDLCs only begin to store energy when low frequencies are reached, but as suggested by the capacitance retention by the [Pyr_1,4_][B(CN)_4_] EDLC in GCD (see Figure 3c), this device starts to store energy at higher frequencies than its counterparts. This effect is also evident considering the relaxation time constant calculated from the peak in Figure 4b, where [Pyr_1,4_][B(CN)_4_] showed a relaxation time constant of 1.9 s while [Pip_1,4_][B(CN)_4_] and [Pyr_1,4_][Tf_2_N] presented the same relaxation time constant of 3.7 s.

**Figure 4:**
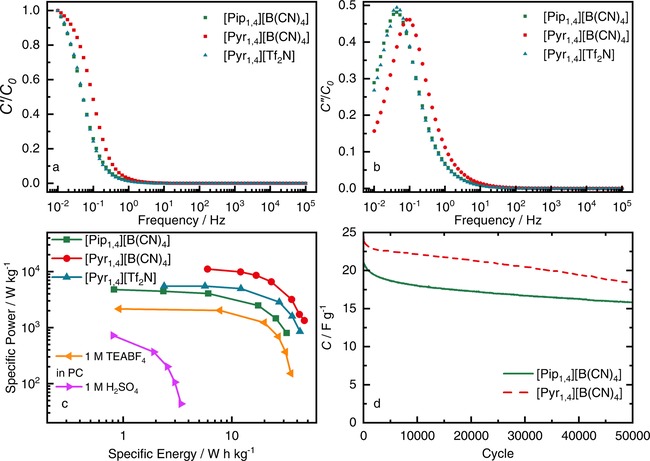
a) Real and b) imaginary part of capacitance versus frequency calculated from EIS spectra for EDLCs containing [Pip_1,4_][B(CN)_4_] (green squares), [Pyr_1,4_][B(CN)_4_] (red circles) and [Pyr_1,4_][Tf_2_N] (blue triangles); Nyquist plot is shown in Figure S2; EIS performed at discharge state (0 V vs. OCV). c) Ragone plot of EDLCs containing [Pip_1,4_][B(CN)_4_] (green squares), [Pyr_1,4_][B(CN)_4_] (red circles) and [Pyr_1,4_][Tf_2_N] (blue triangles), average power and energy values obtained from GCD at different currents, from 0 to 3.7 V, considering only the active mass of both electrodes (symmetrical cells using 1 mol L^−1^ H_2_SO_4_ in water (@ 1.0 V) and 1 mol L^−1^ TEABF_4_ in PC (@ 3.0 V) are shown alongside for comparison). d) Discharge specific capacitance evolution over extended cycles for EDLCs containing [Pip_1,4_][B(CN)_4_] (green full line), [Pyr_1,4_][B(CN)_4_] (red dashed line), operating at 2.0 A g^−1^ from 0 to 3.7 V.

The superior performance of [Pyr_1,4_][B(CN)_4_] EDLCs is evident in the Ragone plot in Figure 4c, which shows the values of specific energy (*E*) and specific average power (*P*_average_) that were calculated from GCD considering only the active mass of both electrodes using the following equations: $E=i\int V/m\cdot 3.6\;dt_{t}$ and $P_{average}=E\cdot 3600/t_{d}$ , where *i*, *V*, *m* and *t_d_* are current (A), voltage (V), the active mass of both electrodes (kg) and discharge time (s). While [Pip_1,4_][B(CN)_4_] shows lower energy and power than [Pyr_1,4_][Tf_2_N], the [Pyr_1,4_][B(CN)_4_] EDLC delivers much higher power than its analogues and outperformed [Pyr_1,4_][Tf_2_N] over the whole studied range. When operating at 11 kW kg^−1^, the [Pyr_1,4_][B(CN)_4_] EDLC can store up to 6 W h kg^−1^, and the [Tf_2_N] IL storing the same amount of specific energy can deliver a specific average power of only 5.7 kW kg^−1^. Moreover, the [Pyr_1,4_][B(CN)_4_] EDLC stores more energy than the [Tf_2_N] EDLC at each specific current. The average specific power delivered by these cells are considerably lower than reported for others ILs in different cell designs,^18^,^44^, ^45^, ^46^ further optimization in order to decrease the ESR is still necessary to improve the power delivery. The cycle life of both [B(CN)_4_] EDLCs were also evaluated cycling between 0 and 3.7 V, and the specific capacitance evolution up to 50000 cycles at 2.0 A g^−1^ are shown in Figure 4d. Both [B(CN)_4_] liquids showed good extended life, with only 12 % and 9 % of specific capacitance decay in the first 5000 cycles, for [Pyr_1,4_][B(CN)_4_] and [Pip_1,4_][B(CN)_4_], respectively, and retained more than 75 % of initial capacitance after 50000 cycles. In addition, Figure S5, in SI, shows the coulombic efficiency of the cells with cycling. Both EDLCs have coulombic efficiency higher than 99.8 % over the 50000 cycles.

In summary, we investigated the performance of two ILs containing the [B(CN)_4_] anion combined with [Pip_1,4_] and [Pyr_1,4_] cations as EDLC electrolytes. These ILs were chosen because they possess greater operating voltage than PC based electrolytes but a greater ionic conductivity than commonly studied ILs (e. g. [Pyr_1,4_][Tf_2_N]). Both ILs possess a wide maximum operating voltage (3.7 V) in comparison with other cyano based anions (in the range of 3.0 V), and comparable to that associated with [Pyr_1,4_][Tf_2_N]. The EDLC assembled with [Pyr_1,4_][B(CN)_4_] demonstrated the highest capacitance and greater capacitance retention, even when operated at high rates. The Ragone plot shows that [Pyr_1,4_][B(CN)_4_] can operate at much higher powers and deliver more energy than the [Pyr_1,4_][Tf_2_N] EDLC at all currents studied. It is important to point out that all EDLCs were assembled with excess of electrolyte (150 μL of IL) so further optimization could improve the volumetric performance of [B(CN)_4_] EDLCs even more. Considering the same volume of IL is needed for each EDLC, the contribution of electrolyte to the device mass would be 40 % lower when the [B(CN)_4_] IL is used.

## **Experimental Section**

The preparation of tetracyanoborate containing ILs has been described elsewhere.^27^,^32^,^47^,^48^ The IL [Pyr_1,4_][Tf_2_N] and the precursors [Pip_1,4_][Br] and [Pyr_1,4_][Br] were purchased from Io−Li‐Tec GmbH (>99 %, Germany). All ILs were stored, handled and dried under vigorous agitation at 100 °C inside an Ar‐filled glovebox (MBraun, H_2_O<0.1 ppm, O_2_<0.1 ppm). ILs were used only after moisture content was lower than 10 ppm, determined by coulometric Karl‐Fischer titration (KF899 Coulometer, Metrohm).

Electrodes were prepared using activated carbon (AC), conductive carbon (Super C45, Imerysys G&C) and PTFE binder (Teflon 30‐N, 60 % suspension in water, Alfa‐Aesar). AC physical properties can be seen in Figure S3, Figure S4 and Table S2 (SI). The AC has a specific surface area (S_BET_) of 1,930 m^2^ g^−1^, arising mainly from micropores and has particles ranging from 1 to 10 μm, with an average size of 5.8 μm. The three materials were blended in a proportion of 80–10‐10 by mass (AC‐C45‐PTFE) in ethanol and mixed using a spatula until a dough‐like consistency was observed. The mixture was then compressed to the desired thickness using a calendering mill, punched into 12 mm discs and dried at 80 °C under vacuum overnight before weighing and cell assembly. Self‐standing electrodes used in EDLCs had thicknesses from 100 to 250 μm and mass loadings from 1.0 to 4.5 mg cm^−2^. Highly asymmetrical electrodes used in maximum operating voltage determinations had thicknesses from 50 to 300 μm.

Coin cells (2016, stainless steel) were assembled inside the glovebox using stainless steel spacers, the electrodes and glass fiber separator (GF/F, Whatman) soaked with excess of IL. In order to improve electrolyte impregnation into electrodes, the cells were kept in the glovebox anti‐chamber under vacuum at 50 °C for five minutes prior to crimping.

The maximum operating voltage of each IL were determined by cyclic voltammetry (CV) at 5 mV s^−1^, using a Solartron Analytical 1470E Multichannel Potentiostat/Galvanostat. Positive and negative limits were determined using fresh cells, which contained a large counter‐electrode (with same composition as the working electrode, *m*_CE_≥20 *m*_WE_). Five cycles were carried out from 0 to 0.5 V (*vs* OCV), for positive determination, or −1.0 V, for negative determination Then, four cycles were recorded before increasing the window by 0.1 V up to 3.0 V, for positive determination, or −3.0 V, for negative determination. The quantity of charge passed during the charge and discharge processes was calculated (q=∫i·dt ) for the last cycle at each window, and the second derivative of the difference of charge (d^2^Δq/dV^2^) was used to determine both the positive and negative limits – a sharp increase in d^2^Δq/dV^2^ indicates the presence of faradaic reactions and a decrease in efficiency.

CV was performed in coil cells using the same set up described above, cycling from 0 V to the determined maximum operating voltage (*vs* OCV), at different scan rates, from 5 to 200 mV s^−1^. Galvanostatic charge‐discharge cycles were carried out at different rates, from 0.5 to 15 A g^−1^, considering the active mass of both electrodes, using a Maccor 4000 M system. 20 cycles were recorded at each rate. The cycle life was evaluated at 2.0 A g^−1^ for 50000 cycles. Electrochemical impedance spectroscopy (EIS) was performed at OCV using a Modulab XCM (Solartron) connected to the Maccor system. A 10 mV perturbation was applied in a frequency range from 100 kHz to 10 mHz, recording 11 points per decade. EIS data were fitted using the Z‐View package. All electrochemical tests were carried out in temperature control chambers at 25 °C (±0.1 °C).

## Conflict of interest

The authors declare no conflict of interest.

## Supporting information

As a service to our authors and readers, this journal provides supporting information supplied by the authors. Such materials are peer reviewed and may be re‐organized for online delivery, but are not copy‐edited or typeset. Technical support issues arising from supporting information (other than missing files) should be addressed to the authors.

Supplementary
