# Supplementary material for: Improved Performance of Ionic Liquid Supercapacitors by using Tetracyanoborate Anions
Source: ChemElectroChem. 2018 Jan 4;5(4):598–604. doi: 10.1002/celc.201701164 (PMC5861663; doi:10.1002/celc.201701164)
Supplement: Supplementary file 1 — Supplementary [file CELC-5-598-s001.pdf]

Supporting Information

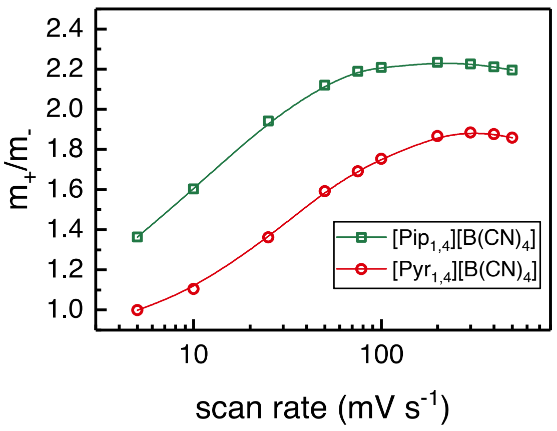

Figure S1. Positive/negative electrode mass balance ratio determined at different scan rates for cells containing $[Pip_{1,4}][B(CN)_4]$ (green squares) and $[Pyr_{1,4}][B(CN)_4]$ (red circles).

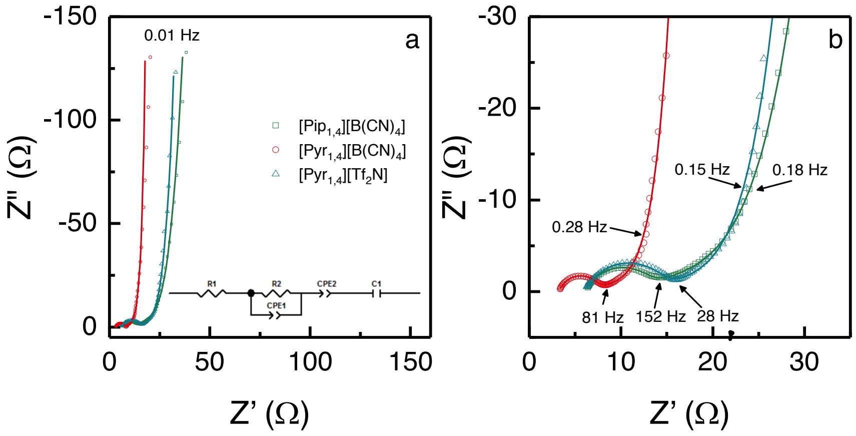

Figure S2. EIS spectra (a – full range, b – high frequency semi-circle) of EDLCs containing $[Pip_{1,4}][B(CN)_4]$ (green line and squares), $[Pyr_{1,4}][B(CN)_4]$ (red line and circles) and $[Pyr_{1,4}][Tf_2N]$ (blue line and triangles). Symbols are experimental data and lines are the best fit for the model in (a).

Table S1. Best fit parameters for EIS spectra. See model in Figure S2a

	R1 (Ω)	R2 (Ω)	CPE1-T (10^{-5} F)	CPE1-P	CPE2-T (F)	CPE2-P	C1 (F)
$[Pip_{1,4}][B(CN_4)]$	5.6±0.05	5.7±0.1	6.4±0.5	0.80±0.009	0.07±0.0005	0.22±0.005	0.13±0.001
$[Pyr_{1,4}][B(CN)_4]$	2.9±0.05	3.6±0.08	3.7±0.4	0.87±0.01	0.14±0.002	0.21±0.008	0.13±0.001
$[Pyr_{1,4}][Tf_2N]$	5.7±0..05	7.5±0.14	8.3±0.7	0.78±0.009	0.09±0.002	0.22±0.007	0.14±0.0007

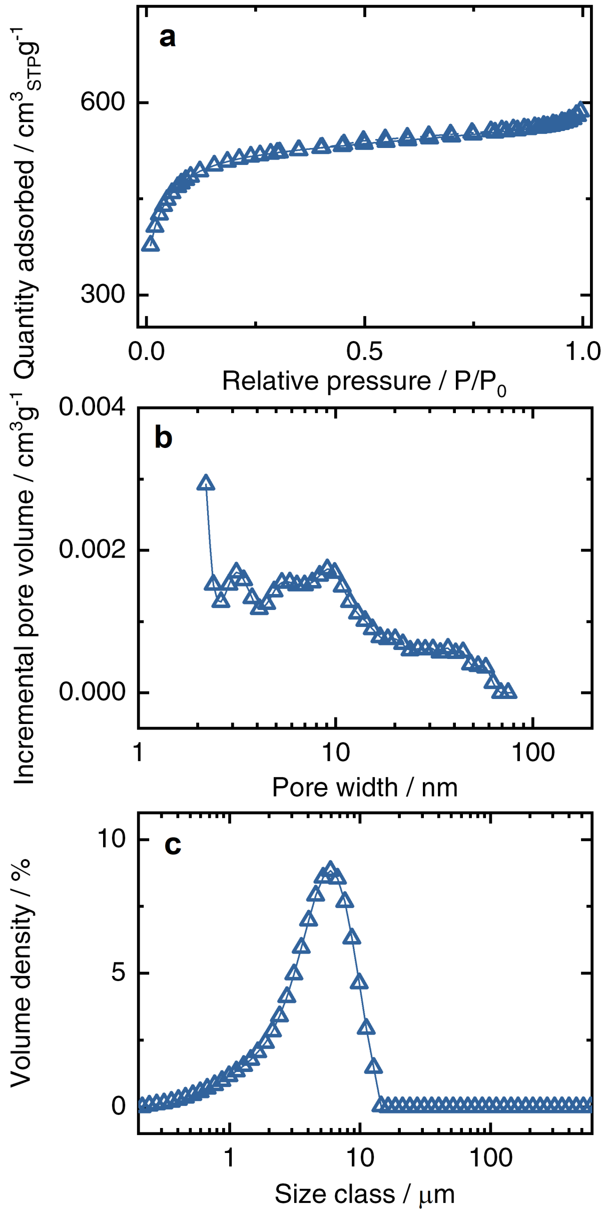

Figure S3. Carbon physical properties. a. Nitrogen adsorption/desorption isotherm at -196 ºC, b. pore size distribution and c. particle size distribution.

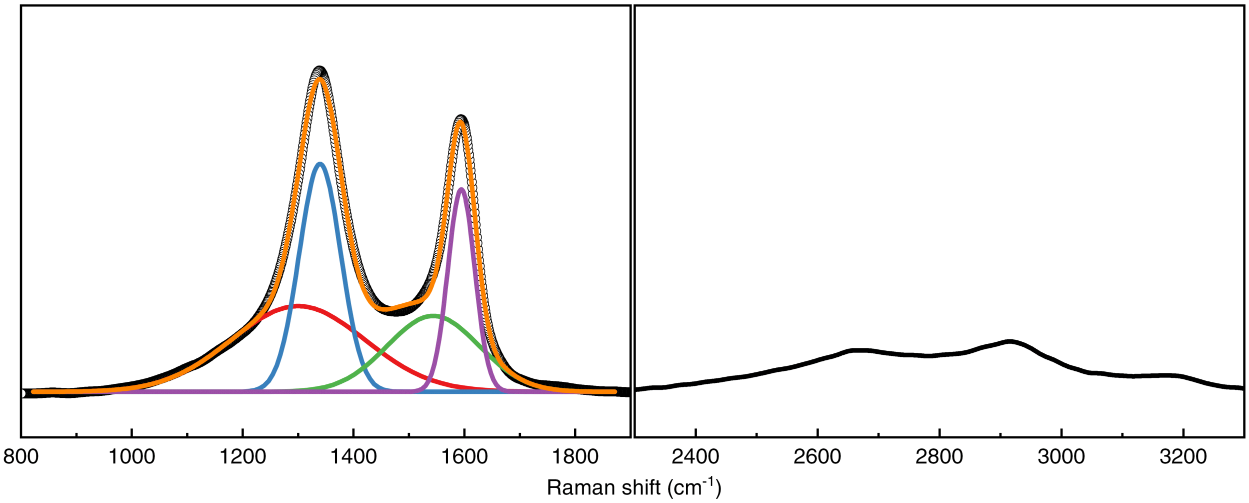

Figure S4. Activated Carbon Raman spectroscopy. Black open symbols (left panel) and black line (right panel) are experimental spectrum, red, blue, green and purple are fitted bands, and orange is the sum of fitted bands.

Table S2. Physical properties of carbon used as active material.

S_{BET}[a] [$m^2\ g^{-1}$]	S_{mic}[b] [$m^2\ g^{-1}$]	S_{meso}[c] [$m^2\ g^{-1}$]	V_t[d] [$cm^3\ g^{-1}$]	V_{mic}[e] [$cm^3\ g^{-1}$]	V_{meso}[f] [$cm^3\ g^{-1}$]
1,930	1,590	161	0.88	0.64	0.15

[a]specific surface area calculated using the BET method [b] micropore surface area determined using the *t*-plot method [c] mesopore surface area determined using BJH method [d]total pore volume calculated at P/P_0>0.95 [e]micropore volume determined using the *t*-plot method[f] mesopore volume determined using BJH method

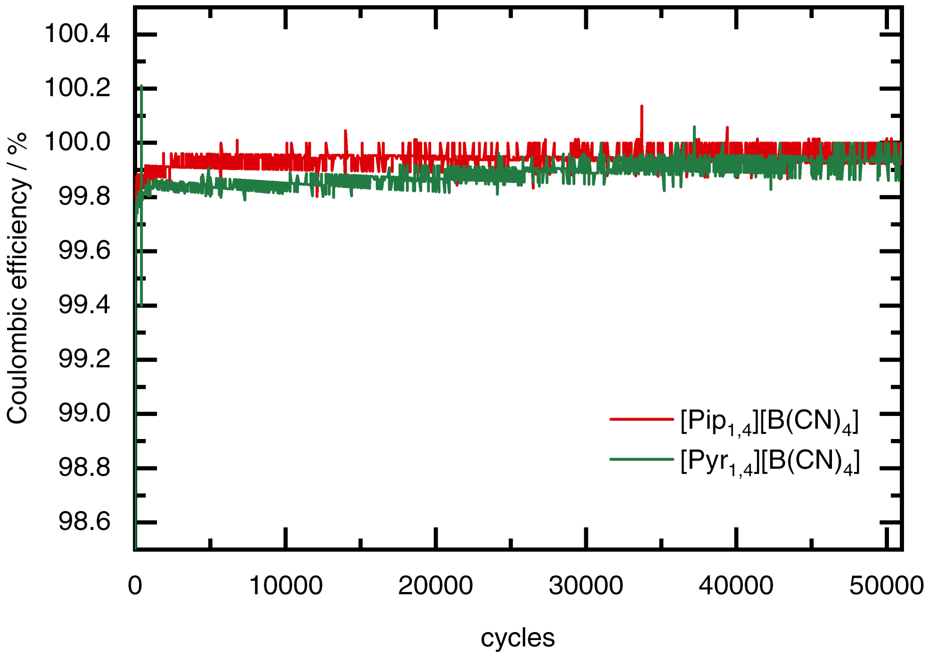

Figure S5. Coulombic efficiency of charge-discharge cycling over 50000 cycles, operating at 2.0 A g^{-1}, from 0 to 3.7 V.
